# Efficacy and safety of sintilimab in combination with chemotherapy in previously untreated advanced or metastatic nonsquamous or squamous NSCLC: two cohorts of an open-label, phase 1b study

**DOI:** 10.1007/s00262-020-02738-x

**Published:** 2020-10-17

**Authors:** Haiping Jiang, Yulong Zheng, Jiong Qian, Chenyu Mao, Xin Xu, Ning Li, Cheng Xiao, Huan Wang, Lisong Teng, Hui Zhou, Shuyan Wang, Donglei Zhu, Tao Sun, Yingying Yu, Wenying Guo, Nong Xu

**Affiliations:** 1 Department of Medical Oncology, The First Affiliated Hospital, School of Medicine, Zhejiang University, No. 79 Qingchun Road, Hangzhou, 310003 China; 2 Department of Surgical Oncology, The First Affiliated Hospital, School of Medicine, Zhejiang University, Hangzhou, China; 3 Department of Medical Science and Strategy Oncology, Innovent Biologics, Inc, Suzhou, China; 4 Hangzhou ImmuQuad Biotechnologies, Hangzhou, China; 5 Zhejiang-California International NanoSystems Institute, Zhejiang University, Hangzhou, China

**Keywords:** Sintilimab, NSCLC, First line, TCR, TMB

## Abstract

Combining chemotherapy with immunotherapy improves the therapeutic outcome for first-line (1L) patients with advance nonsmall-cell lung cancer (NSCLC). Two cohorts of a phase 1b study (NCT02937116) aimed to evaluate the safety and efficacy of sintilimab, a PD-1 inhibitor, plus chemotherapy in 1L patients with nonsquamous and squamous NSCLC (nsqNSCLC/sqNSCLC); and to identify potential biomarkers for treatment response. Treatment-naïve patients with nsqNSCLC were enrolled and intravenously given sintilimab (200 mg), pemetrexed (500 mg/m^2^), and cisplatin (75 mg/m^2^), every 3 weeks (Q3W) for 4 cycles in cohort D. Treatment-naïve patients with sqNSCLC were enrolled and intravenously given sintilimab (200 mg), gemcitabine (1250 mg/m^2^), and cisplatin (75 mg/m^2^), Q3W, for 6 cycles in cohort E. The primary objective was to evaluate the safety and efficacy of the treatment. The additional objective was to explore biomarkers for the treatment efficacy. Twenty-one patients with nsqNSCLC, and 20 patients with sqNSCLC were enrolled in cohort D and cohort E, respectively. By the data cutoff (April 17, 2019), 8 (38.1%) patients in cohort D and 17 (85.0%) patients in cohort E experienced grade 3–4 adverse events. The median follow-up duration was 16.4 months (14.8–23.0) in cohort D and 15.9 months (11.7–17.7) in cohort E. The objective response rate was 68.4% (95% CI 43.4%, 87.4%) in cohort D and 64.7% (95% CI 38.3%, 85.8%) in cohort E. Neither PD-L1 expression nor tumor mutation burden value was significantly associated with an improved treatment response. Sintilimab plus chemotherapy exhibited manageable toxicity and an encouraging antitumor activity in patients with nsqNSCLC and sqNSCLC.

**Electronic supplementary material:**

The online version of this article (10.1007/s00262-020-02738-x) contains supplementary material, which is available to authorized users.

## Introduction

Lung cancer is the leading cause of cancer-related mortality worldwide [1]. Nonsmall-cell lung cancer (NSCLC) represents 85% of the diagnosed lung cancer cases [2], and approximately 70% of the patients with NSCLC are nonsquamous at a diagnosis stage [3].

Platinum-based chemotherapy is the standard treatment for 1L advanced patients with NSCLC lacking targetable mutations [4, 5]. However, the overall clinical outcomes are undesirable. The immunogenic properties of chemotherapies make it possible to combine chemotherapy with immunotherapy [6]. Pembrolizumab combining with platinum-based doublet chemotherapy (PT-DC) has shown an encouraging antitumor activity and manageable toxicity for 1L advanced NSCLC [5, 7].

The biomarkers generated by the treatment response can be used to select patient. PD-L1 is a potential biomarker to predict the response to pembrolizumab monotherapy in 1L metastatic patients with NSCLC harboring PD-L1 tumor proportion score (TPS) ≥ 1% [8]. However, nivolumab showed an inconsistent result [9]. Regarding the combination therapies of PD-1 inhibitors (pembrolizumab or nivolumab) with chemotherapy in 1L setting for patients with NSCLC, there was not a clear association between treatment efficacy with PD-L1 expression [5–7, 10].

Tumor mutation burden (TMB) has emerged as a novel biomarker to identify patients for immunotherapy [11]. In pembrolizumab or nivolumab monotherapy settings, patients with NSCLC and having a high TMB level showed an improved response and survival benefits [12, 13]. Nevertheless, results are discrepant in the combination settings of these PD-1 inhibitors plus chemotherapy as 1L treatment [14, 15].

T cell receptor (TCR) plays significant roles in antigen recognition with the main variable region of complementarity determining region 3 (CDR3) [16]. TCR diversity and clonality are suggested to indicate the clinical outcomes among immune checkpoint inhibitor (ICPi)-treated patients, but controversial results exist among different solid tumors [17, 18]. In addition, studies are scarce in patients received the combination of a PD-1 inhibitor with chemotherapy.

Sintilimab is a highly selective anti-PD-1 antibody with a higher binding affinity to human PD-1 than pembrolizumab and nivolumab [19]. Our Phase 1a study indicated an acceptable safety profile without dose-limiting toxicities in solid tumors. The present phase 1b study described results from two cohorts on NSCLC, aiming to: (1) evaluate the safety and efficacy of the sintilimab plus chemotherapy (sintilimab-chemo) in previously untreated patients with nonsquamous or squamous NSCLC (nsqNSCLC/sqNSCLC) and (2) identify potential TMB or TCR biomarkers for this regimen. Previous results by the data cutoff date on Jan 15, 2019 was reported in 2019 ASCO [20]. Here, we updated the data at cutoff date on Apr 17, 2019 and performed biomarker analyses to provide more evidence for the combination therapy in 1L patients with nsqNSCLC/sqNSCLC.

## Patients and methods

### Inclusion and exclusion criteria

Eligible patients were aged between 18 and 70 years; had histologically or cytologically confirmed nsqNSCLC (cohort D)/sqNSCLC (cohort E) that was unresectable, locally advanced, relapsed, or metastatic; at stage III or IV; and were previously untreated. The staging was based on the 7th edition of the TNM Classification of the International Association for Lung Cancer. Other major inclusion criteria were life expectancy of ≥ 12 weeks and at least one measurable lesion at baseline per Response Evaluation Criteria in Solid Tumors, version 1.1 (RECIST v1.1), Eastern Cooperative Oncology Group performance status (ECOG PS) of 0 or 1 and adequate organ function.

Patients with epidermal growth factor receptor (*EGFR*) mutation or anaplastic lymphoma kinase (*ALK*) rearrangement were excluded from the study. Detailed inclusion and exclusion criteria are available in the Supplementary Materials.

The study was approved by the independent ethics committee at each site and conducted following the Declaration of Helsinki and Good Clinical Practice guidelines. All patients provided written informed consent.

### Study design

The cohort D/E were selected from a multi-center, open-label, phase 1b study including six cohorts (NCT02937116). The D/E cohort was designed to preliminarily assess the efficacy and safety of sintilimab-chemo in patients with nsq/sqNSCLC.

In cohort D, patients received up to 4 cycles of sintilimab (200 mg, IV) in combination with pemetrexed (500 mg/m^2^, IV, D1) and cisplatin (75 mg/m^2^, IV, D1) every 3 weeks. In cohort E, patients received up to 6 cycles of sintilimab in combination with gemcitabine (1250 mg/m^2^, IV, D1) and cisplatin (75 mg/m^2^, IV, D1) every 3 weeks. After combination therapy, patients received sintilimab maintenance therapy (sintilimab plus pemetrexed in cohort D, and sintilimab monotherapy in cohort E) as prior doses every 3 weeks for up to 24 months.

### Endpoints and assessments

The responses to therapy were evaluated at baseline and every 9 weeks after initial dose, using computed tomography (CT) or magnetic resonance imaging (MRI) by the investigator per *RECIST v1.1*. For patients achieved an initial complete remission (CR) or partial remission (PR), the response was confirmed by radiographic evaluation after 4 weeks, and thereafter was assessed every 9 weeks until progression. Safety was assessed mainly by monitoring adverse events (AEs) throughout the trial. AEs were graded in severity based on the National Cancer Institute Common Terminology Criteria (NCICTC version 4.03) guidelines.

The primary endpoints were objective response rate (ORR), defined as the proportion of patients who achieved a CR or PR; and safety profile. The secondary endpoints included PFS, OS, disease control rate (DCR), duration of response (DOR) and time to response (TTR). The exploratory endpoint was the potential correlation between the biomarkers, such as PD-L1, TMB or TCR, and the clinical efficacy of the combination therapy in patients with nsq/sqNSCLC.

### PD-L1 immunohistochemical assay and scoring

PD-L1 immunohistochemical was detected with the Dako PD-L1 IHC 22C3 pharmDx, on the Autostainer Link 48 (clone 22C3, Dako, Carpinteria, CA). Details are provided in the supplementary materials. PD-L1 protein staining was determined by the TPS, which was calculated as the percentage of tumor cells staining with PD-L1 (0–100%). TPS ≥ 1% was defined as PD-L1 positive.

### Tumor mutation burden analysis

The formalin-fixed paraffin-embedded tumor tissues and the matched peripheral blood samples were collected from patients at baseline. After DNA extraction and shearing into fragments, the DNA libraries were constructed with a designed gene panel. Then, the samples were performed with paired-end sequencing on the Illumina HiSeq X-Ten platform (Illumina, San Diego, USA). TMB was measured in mutations per one million coding bases (Mb), including somatic single-nucleotide variants and indel mutations in the whole exome. The detailed sequencing methods are provided in supplementary materials. Patients were divided into TMB-high (TMB-H) and TMB-low (TMB-L) groups based on the median TMB cutoff.

### Analysis of TCR repertoire

Total RNA of total CD8^+^ T cells were extracted using the RNeasy Plus Mini Kit (Qiagen, Valencia, CA, USA) according to the manufacture’s instruction. Then, RNA samples were performed with high-throughput sequencing of TCR using the ImmuHub^®^ *TCR* profiling system at a deep level (ImmuQuad Biotech, Hangzhou China), as described in the supplementary methods and our previous reports [21, 22]. Shannon’s index of diversity and clonality index were calculated using formulas described in the supplementary methods.

### Statistical analyses

Twenty patients were planned to enroll in each cohort (D or E). Patients who received 1 or more drug dose were enrolled in the efficacy and safety analysis sets. ORR and DCR were estimated using the binomial distribution, and the two-sided 95% exact confidence intervals (CIs) were calculated by the Clopper–Pearson method. The time-to-event endpoints (median PFS, OS, DOR, TTR, and PFS and OS rates at 6 and 12 months) were assessed by Kaplan–Meier product–limit method. The survival curves were estimated by the log-rank test.

Student’s *t* test (two-sided) was used to evaluate the TCR diversity and clonality index pre- and post-treatment, by GraphPad Prism version 6.0 (La Jolla, CA, USA). Other statistical analyses were conducted with the SAS software (version 9.2 or higher). *P* < 0.05 was the cutoff for significance.

## Results

### Patients characteristics

Baseline characteristics of patients in the two cohorts are presented in Table 1. From May 9, 2017 to January 15, 2018, 21 previously untreated patients with nsqNSCLC were enrolled in cohort D, with a median age of 62.6 (55.8–65.9) years. At baseline, 16 (76.2%) patients had stage IV disease, 7 (33.3%) were nonsmokers, and 19 (90.5%) had ECOG PS of 1 (Table 1). At the data cutoff date, April 17, 2019, 13 (61.9%) patients discontinued treatment mainly due to disease progression (7/13, 53.8%). The median follow-up duration was 16.4 months (range 14.8–23.0).

**Table 1:** Baseline Characteristics of patients receiving sintilimab in combination with chemotherapy in two cohorts

	Cohort D (*n* = 21)	Cohort E (*n* = 20)
Age, median (range), years	62.6 (55.8–65.9)	65.0 (60.2–68.7)
Sex		
Male	16 (76.2%)	19 (95.0%)
Female	5 (23.8%)	1 (5.0%)
ECOG PS		
0	2 (9.5%)	9 (45.0%)
1	19 (90.5%)	11 (55.0%)
Disease stage		
IIIB	4 (19.0%)	9 (45.0%)
IIIC	1 (4.8%)	0
IV	16 (76.2%)	11 (55.0%)
Histology		
Adenocarcinoma	21 (100%)	0
Squamous cell carcinoma	0	20 (100%)
Smoking status		
Current	2 (9.5%)	2 (10.0%)
Former	12 (57.1%)	15 (75.0%)
Never	7 (33.3%)	3 (15.0%)
Prior treatments of bone metastasis	1 (4.8%)	2 (10.0%)

*ECOG PS* Eastern Cooperative Oncology Group performance status

From October 17, 2017 to April 18, 2018, Cohort E enrolled 20 treatment-naïve patients with sqNSCLC, with a median age of 65.0 (60.2–68.7) years. At baseline, 11 (55.0%) patients had stage IV disease, 3 (15.0%) were nonsmokers, and 11 (55.0%) had ECOG PS of 1 (Table 1). At the time of analysis, a majority of patients terminated treatment (17/20, 85%), and most of them (10/17, 58.8%) were because of disease progression. The median follow-up duration was 15.9 months (range 11.7-17.7).

Patients received a median of 13.0 doses (range 1–26) and 9.0 doses (4–16) of sintilimab in cohort D (*n* = 21) and cohort E (*n* = 20), respectively; and the median duration of exposure to the treatments was 39.3 weeks (range 0.1–75.4) and 27.1 weeks (9.1–45.1), respectively.

### Safety

In cohort D (*n* = 21), AEs of any grade were reported in 19 (90.5%) patients, including the most frequent neutrophil count decreased (10, 47.6%), nausea (9, 42.9%), and anemia (9, 42.9%). Eight (38.1%) patients occurred grade 3 or 4 AEs, with lung infection (2, 9.5%) and rash (2, 9.5%) most commonly. Fifteen (71.4%) patients experienced sintilimab-related AEs, including alanine aminotransferase (ALT) increased (5, 23.8%) and fatigue (5, 23.8%). Sintilimab-related AEs of grades 3 or 4 were reported in 3 (14.3%) patients, each experiencing blood triglyceride elevated, functional gastrointestinal disorder and rash, respectively. Immune-related AEs (irAEs) per investigator were recorded in 6 (28.0%) patients; most of the irAEs were grades 1–2, and only one event was grade 3 (rash). No grade 5 AEs or AEs-led death were reported (Table 2). Only one (4.8%) patient experienced AEs that leading to permanent discontinuation.

**Table 2:** Adverse events (AEs) reported in ≥ 10% patients or ≥ grade 3 AEs receiving sintilimab in combination with chemotherapy in cohort D or cohort E

No. of patients (%)								
	Cohort D (*n* = 21)				Cohort E (*n* = 20)			
	Any grade	Grade 1–2	Grade 3	Grade 4	Any grade	Grade 1–2	Grade 3	Grade 4
All events	19 (90.5)	11 (52.4)	6 (28.6)	2 (9.5)	20 (100.0)	3 (15.0)	13 (65.0)	4 (20.0)
Neutrophil count decreased	10 (47.6)	10 (47.6)	0	0	16 (80.0)	6 (30.0)	8 (40.0)	2 (10.0)
Nausea	9 (42.9)	9 (42.9)	0	0	8 (40.0)	6 (30.0)	2 (10.0)	0
Anemia	9 (42.9)	8 (38.1)	1 (4.8)	0	17 (85.0)	10 (50.0)	7 (35.0)	0
Decreased appetite	8 (38.1)	8 (38.1)	0	0	9 (45.0)	8 (40.0)	1 (5.0)	0
WBC count decreased	7 (33.3)	7 (33.3)	0	0	18 (90.0)	10 (50.0)	8 (40.0)	0
ALT elevated	7 (33.3)	7 (33.3)	0	0	1 (5.0)	1 (5.0)	0	0
Fatigue	7 (33.3)	7 (33.3)	0	0	5 (25.0)	4 (20.0)	1 (5.0)	0
Vomiting	7 (33.3)	7 (33.3)	0	0	6 (30.0)	5 (25.0)	1 (5.0)	0
AST elevated	6 (28.6)	6 (28.6)	0	0	2 (10.0)	2 (10.0)	0	0
Pyrexia	6 (28.6)	6 (28.6)	0	0	6 (30.0)	6 (30.0)	0	0
Rash	5 (23.8)	3 (14.3)	2 (9.5)	0	4 (20.0)	4 (20.0)	0	0
GGT elevated	4 (19.0)	3 (14.3)	0	1 (4.8)	4 (20.0)	2 (10.0)	2 (10.0)	0
Constipation	4 (19.0)	4 (19.0)	0	0	2 (10.0)	2 (10.0)	0	0
Diarrhea	4 (19.0)	3 (14.3)	1 (4.8)	0	2 (10.0)	2 (10.0)	0	0
Lung infection	4 (19.0)	2 (9.5)	2 (9.5)	0	1 (5.0)	1 (5.0)	0	0
Insomnia	4 (19.0)	4 (19.0)	0	0	3 (15.0)	3 (15.0)	0	0
Platelet count decreased	2 (9.5)	2 (9.5)	0	0	15 (75.0)	11 (55.0)	2 (10.0)	2 (10.0)
Hypertension	2 (9.5)	2 (9.5)	0	0	6 (30.0)	1 (5.0)	5 (25.0)	0
Hypoalbuminemia	1 (4.8)	1 (4.8)	0	0	5 (25.0)	5 (25.0)	0	0
Proteinuria	1 (4.8)	1 (4.8)	0	0	4 (20.0)	4 (20.0)	0	0
Sintilimab-related AEs	15 (71.4)	12 (57.1)	2 (9.5)	1 (4.8)	13 (65.0)	10 (60.0)	1 (5.0)	0
ALT elevated	5 (23.8)	5 (23.8)	0	0	0	0	0	0
Fatigue	5 (23.8)	5 (23.8)	0	0	0	0	0	0
AST elevated	4 (19.0)	4 (19.0)	0	0	1 (5.0)	1 (5.0)	0	0
Rash	4 (19.0)	3 (14.3)	1 (4.8)	0	2 (10.0)	2 (10.0)	0	0
Vomiting	4 (19.0)	4 (19.0)	0	0	0	0	0	0
Pyrexia	2 (9.5)	2 (9.5)	0	0	2 (10.0)	2 (10.0)	0	0
Blood TG elevated	1 (4.8)	0	0	1 (4.8)	0	0	0	0
Functional gastrointestinal disorder	1 (4.8)	0	1 (4.8)	0	0	0	0	0
Hypothyroidism	1 (4.8)	0	0	0	2 (10.0)	2 (10.0)	0	0
Interstitial lung disease	1 (4.8)	0	0	0	2 (10.0)	2 (10.0)	0	0
Neutrophil count decreased	3 (14.3)	3 (14.3)	0	0	1 (5.0)	0	1 (5.0)	0
Immune-related AEs by investigator	6 (28.6)	5 (23.8)	1 (4.8)	0	5 (25.0)	5 (25.0)	0	0
Rash	4 (19.0)	3 (14.3)	1 (4.8)	0	1 (5.0)	1 (5.0)	0	0
Blood TSH decreased	3 (14.3)	3 (14.3)	0	0	0	0	0	0
Interstitial lung disease	1 (4.8)	1 (4.8)	0	0	2 (10.0)	2 (10.0)	0	0

*WBC* white blood cell, *ALT* alanine aminotransferase, *AST* aspartate aminotransferase, *GGT* Gamma-glutamyltransferase, *TG* triglyceride, *TSH* thyroid-stimulating hormone

In cohort E (*n* = 20), the occurrence of AEs of any grade was 100%, and the most common events were white blood cell (WBC) count decreased (18, 90.0%), anemia (17, 85.0%), and neutrophil count decreased (16, 80.0%). AEs of grade 3 or 4 occurred among 17 (85.0%) patients, most commonly with WBC count decreased (8, 40.0%), neutrophil count decreased (8, 40.0%), and anemia (7, 35.0%). Sintilimab-related AEs were reported in 13 (65.0%) patients, including rash (2, 10.0%), interstitial lung disease (2, 10.0%), hypothyroidism (2, 10.0%), and pyrexia (2, 10.0%); and only one (5.0%) event was grade 3 (neutrophil count decreased). AEs in two patients (10.0%) induced permanent discontinuation. No AEs of grade 5 or more led to death (Table 2).

### Efficacy

**Fig. 1:**
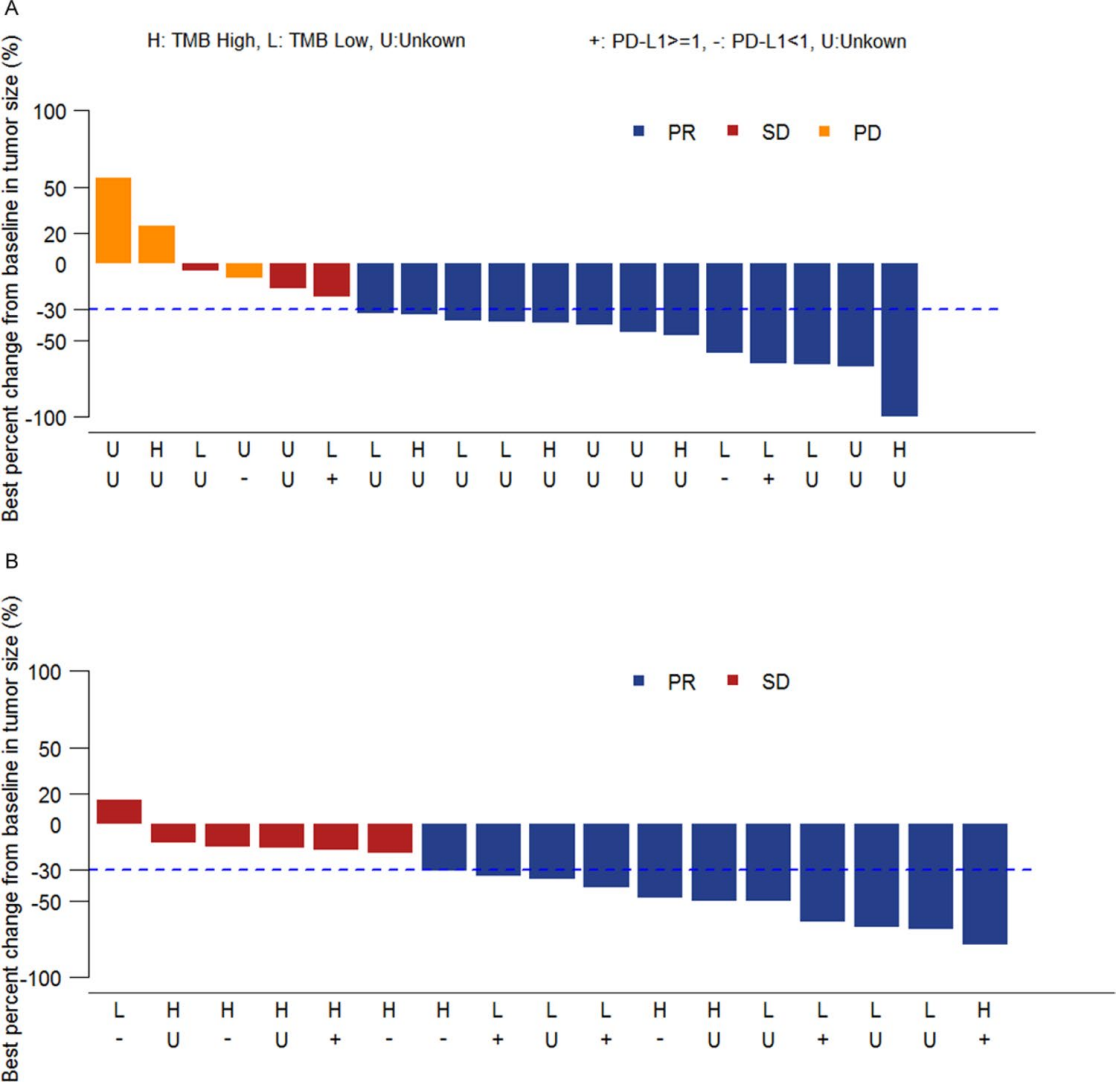
Waterfall plot of the best response to sintilimab-chemo combination therapy. **a** Cohort D, **b** Cohort E. *PR* partial remission, *SD* stable disease, *PD* progressive disease, *TMB* tumor mutation burden

In cohort D, at the data cutoff (April 17, 2019), among 19 evaluable patients who had response assessment for at least once after treatment, 13 patients (68.4%) reached PR, 3 (15.8%) had stable disease (SD), and 3 (15.8%) had progressive disease (PD) (Fig. 1a). Among these 19 patients, the ORR per *RECIST v1.1* was 68.4% (95% CI 43.4%, 87.4%), and DCR was 84.2% (95% CI 60.4%, 96.6%) (Table 3). Among the responders, the continuous response rate (percentage of patients who had a continuous response at the study end) was 61.5% (95% CI 31.6%, 86.1%). The median TTR was 2.1 months (95% CI 2.1, 4.0). The median DOR was not reached (Table 3).

**Table 3:** Antitumor activity of patients in two cohorts

	Cohort D (*n* = 21)^a^	Cohort E (*n* = 20)^b^
Best overall response per RECIST v1.1		
CR (%)	0	0
PR (%)	13 (68.4)	11 (64.7%)
SD (%)	3 (15.8)	6 (35.3%)
PD (%)	3 (15.8)	0
ORR (CR + PR), n % [95% CI]	13 (68.4) [43.4, 87.4]	11 (64.7) [38.3, 85.8]
DCR (CR + PR + SD), n % [95% CI]	16 (84.2) [60.4, 96.6]	17 (100.0) [80.5, 100.0]
Median TTR, months (95% CI)	2.1 (2.1, 4.0)	2.1 (1.9, 2.3)
Median DOR, months (95% CI)	NA (5.8, NA)	5.7 (1.9, NA)

*RECIST* Response Evaluation Criteria in Solid Tumors, *CR* complete remission, *PR* partial remission, *SD* stable disease, *PD* progressive disease, *ORR* objective response rate, *DCR* disease control rate, *DOR* duration of response, *TTR* time to response, *CI* confidence interval, *NA* not available

^a^Assessed in 19 evaluable patients in cohort D

^b^Assessed in 17 evaluable patients in cohort E

In cohort E, at the data cutoff (April 17, 2019), 17 patients were evaluable, and 11 (64.7%) achieved PR, 6 (35.3%) had SD, and no patients developed PD (Fig. 1b). ORR per *RECIST v1.1* among the evaluable patients was 64.7% (95% CI 38.3%, 85.8%), and DCR was 100.0% (95% CI 80.5%, 100.0%) (Table 3). Among the responders, the continuous response rate was 45.5% (95% CI 16.7%, 76.6%). The median TTR was 2.1 months (95% CI 1.9, 2.3), and the estimated median DOR was 5.7 months (95% CI 1.9, NA) (Table 3).

**Fig. 2:**
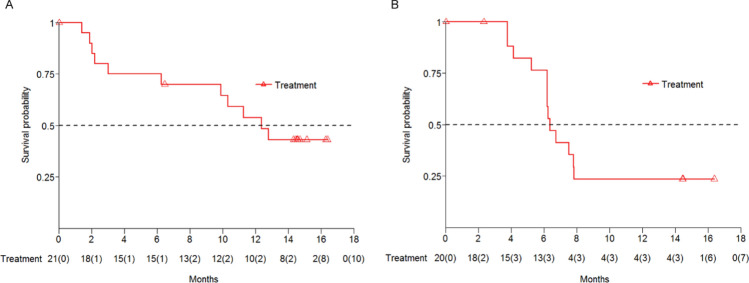
Kaplan-Meier curves of progression-free survival. **a** Cohort D, **b** Cohort E

In cohort D, at the time of analysis, the estimated median PFS by *RECIST v1.1* was 12.6 months (95% CI 3.1, NA), and the PFS rates at 6 and 12 months were 75.0% (95% CI 50.0%, 89.0%) and 54.0% (95% CI 30.0%, 73.0%), respectively (Fig. 2a). The estimated median OS per *RECIST v1.1* was 18.9 months (95% CI 5.3, NA), the OS rates at 6 and 12 months were 75.0% (95% CI 50.0%, 89.0%) and 70.0% (95% CI 45.0%, 85.0%), respectively.

In cohort E, based on *RECIST v1.1*, the median PFS was 6.5 months (95% CI 5.3, 8.0). PFS rate at 6 months was 76.0% (95% CI 49.0%, 90.0%), and at 12 months was 24.0% (95% CI 7.0%, 45.0%) (Fig. 2b). The estimated median OS was 15.4 months (95% CI 10.3, NA). OS rate at 6 months was 84.0% (95% CI 59.0%, 95.0%), and at 12 months was 62.0% (95% CI 36.0%, 80.0%).

### Correlation between PD-L1 expression and response

**Fig. 3:**
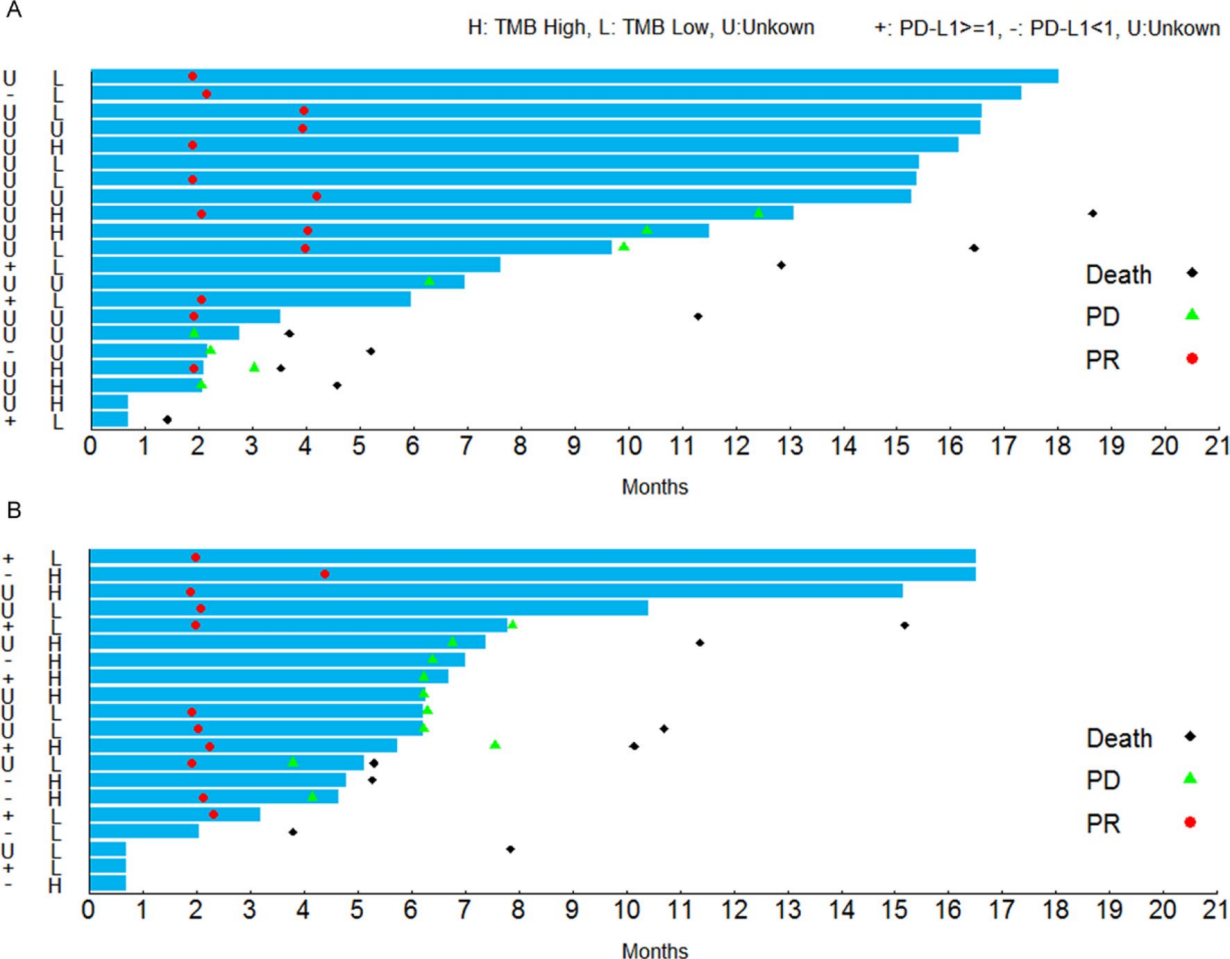
Response and duration for the patients receiving sintilimab-chemo combination therapy with different PD-L1 expressions and TMB values. **a** Cohort D, **b** Cohort E. *PR* partial remission, *PD* progressive disease, *TMB* tumor mutation burden

In cohort D, among 5 patients with evaluable PD-L1 expression, 3 (60%) patients had high PD-L1 with TPS ≥ 1%, and 2 had low PD-L1 level (TPS < 1%) (Fig. 3a). Among these evaluable patients, tumor response had no significant association with PD-L1 expression (TPS ≥ 1% vs. TPS < 1%: ORR 33.3% vs. 50.0%, *P* > 0.05).

In cohort E, PD-L1 expression was quantifiable on 12 patients. Among these patients, 6 patients had high tumor PD-L1 expression (TPS ≥ 1%), and 6 had low tumor PD-L1 expression (TPS < 1%) (Fig. 3b). Likewise, no significant correlations were observed between tumor response and PD-L1 expression (TPS ≥ 1% vs. TPS < 1%: ORR 80% vs. 40%, *P* = 0.5238).

### Correlation between TMB and response

In cohort D, 15 patients had evaluable TMB. 46.7% (7/15) patients were in the TMB-H group (TMB ≥ 4.25), and of them, 6 patients had evaluable tumor assessments with an ORR of 83.3% (5/6, 95% CI 35.9%, 99.6%) (Fig. 3a). 53.3% (8/15) patients were in TMB-L group (TMB < 4.25), and 7 out of the 8 patients had evaluable tumor assessments, achieving an ORR of 71.4% (5/7, 95% CI 29.0%, 96.3%) (Fig. 3a). In cohort D, the patients with a high TMB did not show a significantly better response than those with a low TMB (*P* > 0.05).

In cohort E, all 20 patients had evaluable TMB, 12 (60.0%) patients were in the TMB-H (TMB ≥ 4.25) group and 8 (40.0%) in the TMB-L (TMB < 4.25) group. The ORR among patients with evaluable tumor assessment in the TMB-H (*n* = 11) and TMB-L (*n* = 6) groups were 54.5% (6/11) (95% CI 23.3%, 83.3%) and 83.3% (5/6) (95% CI 35.9%, 99.6%), respectively (Fig. 3b). In cohort E, the patients with a high TMB also did not show a better response than those with a low TMB (*P* = 0.3334).

### TCR repertoire and efficacy

Owing to the small patient sample size, we merged patients in the two cohorts to perform the TCR correlation analysis. At the time of analysis, the patients who had progressed and out of the study were classified into PD group, and the remaining were into disease control (DC) group. Overall, 11 patients had evaluable TCR, 5 in DC group and 6 in PD group.

**Fig. 4:**
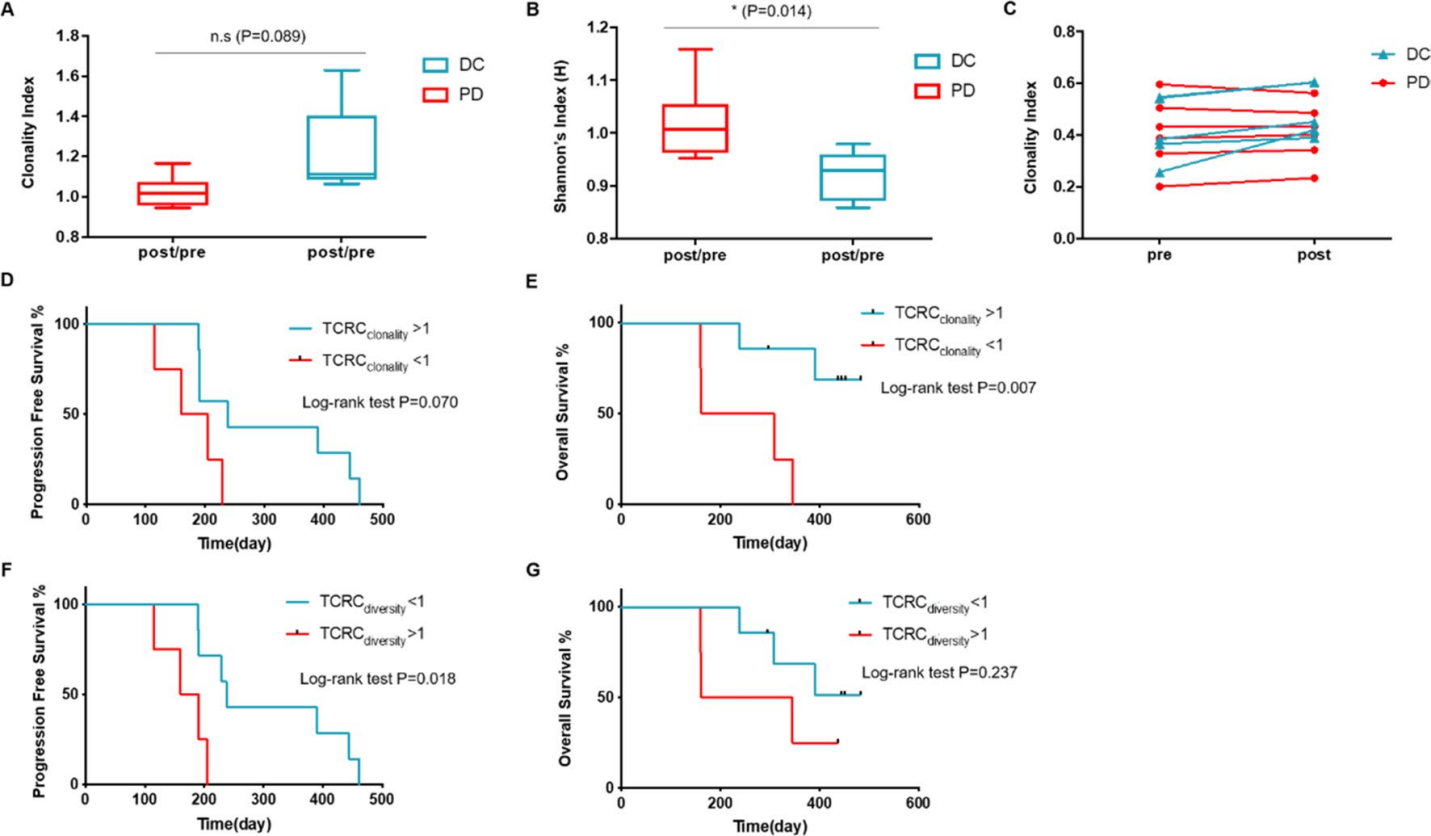
Association of CD8^+^ T cell receptor (TCR) and treatment efficacy. **a** TCRC_clonality_ (the ratio of TCR clonality index post- and pre-treatment) between disease control (DC) group and progressive disease (PD) group; **b** TCRC_diversity_ (the ratio of TCR diversity index post- and pre-treatment) between DC and PD groups; **c** TCR clonality tracking within the treatment of every patient; **d**, **e** progression-free survival (PFS) and overall survival (OS) stratified by TCRC_clonality_ (> 1 vs. < 1); F and G, PFS and OS stratified by TCRC_diversity_ (> 1 vs. < 1)

Patients in DC group showed an increased TCR Change (TCRC) clonality index (TCRC_clonality_), namely the ratio of TCR clonality index post- and pre-treatment, when compared with those in PD group. However, the difference was not statistically significant (*P* = 0.089, Fig. 4a). The TCRC diversity index (TCRC_diversity_, the ratio of TCR diversity index post- and pre-treatment) in the DC group was significantly decreased when compared with the PD group (*P* = 0.014, Fig. 4b). The TCR clonality in the DC group was increased post-treatment when compared with pre-treatment, while that in PD group had an approximately stable change trend (Fig. 4c).

To better reflect the association between the treatment efficacy with the changing trend of TCR, we used the TCRC_clonality_ or TCRC_diversity_ of 1 as cutoff, and found a high TCRC_clonality_ (> 1) was significantly associated with an improved OS (*P* = 0.007, Fig. 4e); in contrast, a low TCRC_diversity_ (< 1) had an evident association with a prolonged PFS (*P* = 0.018, Fig. 4f).

## Discussion

Due to the immunogenic properties of chemotherapy, immunotherapy in combination with chemotherapy (immunotherapy-chemo) approach has been widely studied. Our phase 1b study preliminarily suggested that sintilimab-chemo had a tolerable safety profile and may improve tumor response in 1L advanced patients with nsq/sqNSCLC.

In our study, most frequent AEs for the combination therapy in two cohorts were nonhematological, such as nausea (42.9%) which was comparable to historical data of pembrolizumab- or nivolumab-chemo (29–56%) in advanced patients with NSCLC [5, 6, 23]. Hematological events also occurred in our study, such as anemia (42.9% in cohort D and 85.0% in cohort E), which was similar to previous data of gemcitabine-cisplatin chemotherapy and pembrolizumab-chemo (45–47%) [5, 24]. The occurrences of neutrophil count decreased (80.0% vs. 47.6%) and anemia (85.0% vs. 42.9%) were higher in cohort E (sintilimab plus cisplatin + gemcitabine) than that in cohort D (sintilimab plus cisplatin + pemetrexed). The different occurrences were possibly caused by different chemotherapy regimens. This phenomenon was observed in 1L nivolumab (10 mg/kg) plus the same chemotherapy regimens (17% vs. 0% and 50% vs. 13%, respectively) [6]. Rash was one of the most frequent grade 3 or 4 AEs in cohort D (no more than 10%), consistent with historical data of pembrolizumab (1.7%) [5]. Lung infection was also the frequent AE of grade 3 or 4 (10%), but it was manageable and did not cause any death. In cohort E, the most common AEs were decreased WBC count and decreased neutrophil count (less than 50%), which were also comparable to nivolumab- and pembrolizumab-chemo (less than 20%) settings [5, 6].

In this study, most sintilimab-related AEs were grade 1–2 in severity, such as ALT increased, fatigue, rash, and hypothyroidism, which were also previously reported relating to nivolumab [6]. Except for one patient showing grade 3 immune-related AEs (rash), most immune-related AEs were grade 1–2, mainly affecting the skin and pulmonary organs. No AEs of grade 5 were reported in our study, indicating a tolerable safety profile of this combination strategy.

Combination therapy of PD-1 inhibitors and chemotherapy could enhance the anti-tumor activity of chemotherapy and improve the treatment response. In 1L patients with nsqNSCLC receiving pembrolizumab-chemo (carboplatin and pemetrexed), the ORR was 75% (18/24) [23]. In 1L nivolumab-chemo (PT-DC) treated patients with NSCLC, the confirmed ORR ranged from 33 to 47% across different dose groups [6]. Sintilimab-chemo in our study showed an increased response than nivolumab-chemo, with an ORR of 68.4% (cohort D) or 64.7% (cohort E). Regarding the association between PD-L1 expression and response, the patients with NSCLC had similar ORRs to pembrolizumab-chemo among different PD-L1 TPS groups [23, 25]. In addition, in nivolumab-chemo treated advanced patients with NSCLC, the confirmed ORR was not affected by different expression levels of PD-L1 [6]. Notably, in a phase 3 study, pembrolizumab-chemo showed a superior response benefit over chemotherapy in metastatic patients with nsqNSCLC, regardless of the tumor PD-L1 expression [5]. Consistent with these preliminary results, our study indicated no significant correlations between PD-L1 expression and responses to sintilimab-chemo in both patients with nsqNSCLC and sqNSCLC.

The immunotherapy-chemo strategy also revealed an improved long-term survival benefit. The 1L nivolumab-chemo (PT-DC) had a promising survival benefit for advanced patients with NSCLC, with a median PFS ranging from 4.8 to 7.1 months and a median OS from 11.6 to 19.2 months, both of which were longer than previous data of PT-DC alone [6]. Pembrolizumab-chemo (pemetrexed + cisplatin/carboplatin) significantly prolonged the median PFS when compared with chemotherapy alone for previously untreated metastatic patients with nsqNSCLC lacking *EGFR* or *ALK* mutations (8.8 m vs. 4.9 m) [5]. Our results showed sintilimab-chemo had an improved long-term survival outcome with an estimated median PFS of 12.6 months in patients with nsqNSCLC and 6.5 months in patients with sqNSCLC. Notably, the 12-month OS rate (70.0% or 62.0%) in this study was similar to the historical data of pembrolizumab- or nivolumab-chemo (69.2% or 50–87%, respectively) [5, 6]. However, it could not make an assertive conclusion due to the immature data.

A clonal expansion of the neoantigen-specific T cells is expected after the response to anti-PD-1/PD-L1 regimens [16]. In multiple cancers, increased TCR clonality after ICPs is associated with an improved treatment efficacy [26]. The TCR-Vβ underwent expansions after neoadjuvant immunotherapy with atezolizumab in NSCLC [27]. Increased CD4^+^ TCR repertoire clonality was correlated with a high density of tertiary lymphoid structure B cells, which was a biomarker of a higher OS in NSCLC [28]. Before treatment, high TCR diversity indicates a better immune status, with the mechanism that high diversity could preclude the magnitude of immune escape via increasing more potential tumor-specific T cells, which can control tumor cell growth and recognize corresponding antigens [17]. In patients with NSCLC, those with an increased peripheral PD-1^+^ CD8^+^ (double-positive PD-1 and CD8) clonality after ICPi treatment, and with a high PD-1^+^ CD8^+^ diversity pre-ICPi exhibited a better response and a longer PFS, as compared to those with low clonality and diversity [16]. Consistent with this finding, in our study, the higher TCRC_clonality_ (which reflected an increased TCR clonality post-treatment) or lower TCRC_diversity_ (indicating a high TCR diversity pre-treatment) had an evident association with a prolonged OS or PFS benefit from the sintilimab-chemo treatment. Nevertheless, further evidence is required to support the potential use of these indexes as effective predictors for the combination strategy of PD-1 inhibitor and chemotherapy.

It was suggested that mutational landscape of NSCLCs might affect the response to anti-PD-1 therapy, and TMB might be a promising biomarker for selecting appropriate patients [12, 29]. A high TMB is commonly considered to promote neoantigens formation, and the most mutated tumors are likely to be the most immunogenic ones [30]. Although FDA has accepted a priority review to a supplemental Biologics License Application for pembrolizumab for the 1L treatment of solid tumors among TMB-H (≥ 10 mut/Mb) populations based on Keynote 158 [31], TMB is not the standard biomarker for predicting the efficacy of PD-1/PD-L1 inhibitors. Moreover, the positive correlation between high TMB and the improved response of PD-1 inhibitors was generally acknowledged in the monotherapy [13, 32]. Meanwhile, the prediction of TMB on response to ICPi is inconclusive since the mutation ranges are overlapped between responders and nonresponders [30]. In patients treated with immunotherapy-chemo, inconsistent results also existed on the relationship between TMB and the treatment efficacy. A high TMB (≥ 10 mut/Mb) was associated with a prolonged PFS in 1L nivolumab-chemo-treated patients with advanced NSCLC [14], whereas a high tissue TMB (≥ 175 mut/exome) did not present a significant correlation with the efficacy in 1L pembrolizumab-chemo-treated patients with metastatic nsq/sqNSCLC [15]. In our study, in both nsqNSCLC and sqNSCLC cohorts, patients with a high TMB (≥ 4.25) did not show a significantly better response to sintilimab-chemo.

Despite the promising findings, there were some limitations. The antitumor activity of sintilimab-chemo was reported in a single-arm phase 1 study with a small sample size, and should be proven in large populations. Besides, the biomarker results, such as PD-L1, TMB, and TCR also require further confirmation because of the small patient samples. Meanwhile, very few patients had evaluable TCR, so the interpretations about TCR need to be cautious. Nonetheless, our study provides preliminary evidence for sintilimab-chemo as 1L treatment in patients with advanced nsq/sqNSCLC. Two phase 3 studies are currently ongoing to evaluate the combination therapy in 1L patients with nsqNSCLC (NCT03607539) and sqNSCLC (NCT03629925), respectively.

In conclusion, sintilimab in combination with pemetrexed-cisplatin or with gemcitabine-cisplatin showed manageable toxicity and an encouraging antitumor activity in patients with nsqNSCLC and sqNSCLC, regardless of PD-L1 expression or TMB level. A phase 3 study investigating sintilimab-chemo as 1L treatment in patients with NSCLC, irrespective of PD-L1 expression, is currently ongoing and has achieved endpoint in the interim analysis.

## Electronic supplementary material

Below is the link to the electronic supplementary material.

Supplementary material 1 (DOCX 21 kb)

## Data Availability

To protect patients information, the datasets generated in this clinical study are not public, but they could be available from the corresponding author on request.

## Abbreviation

AEs: Adverse events
ALK: Anaplastic lymphoma kinase
ALT: Alanine aminotransferase
CDR3: Complementarity determining region 3
CT: Computed tomography
CR: Complete remission
DCR: Disease control rate
DOR: Duration of response
ECOG PS: Eastern Cooperative Oncology Group performance status
EGFR: Epidermal growth factor receptor
ICPi: Immune checkpoint inhibitor
Immunotherapy-chemo: Immunotherapy in combination with chemotherapy
MRI: Magnetic resonance imaging
NCICTC: National Cancer Institute Common Terminology Criteria
NSCLC: Advance nonsmall-cell lung cancer
nsqNSCLC: Nonsquamous NSCLC
ORR: Objective response rate
OS: Overall survival
PD: Progressive disease
PD-1^+^ CD8^+^: Double-positive PD-1 and CD8
PFS: Progression-free survival
PR: Partial remission
PT-DC: Platinum-based doublet chemotherapy
RECIST v1.1: Response Evaluation Criteria in Solid Tumors, version 1.1
SD: Stable disease
sqNSCLC: Squamous NSCLC
TCR: T cell receptor
TCRC: TCR change
TCRC_clonality_: TCRC clonality index
TCRC_diversity_: TCRC diversity index
TMB: Tumor mutation burden
TPS: Tumor proportion score
TTR: Time to response
WBC: White blood cell
