# Supplementary material for: Efficacy and safety of sintilimab in combination with chemotherapy in previously untreated advanced or metastatic nonsquamous or squamous NSCLC: two cohorts of an open-label, phase 1b study
Source: Cancer Immunol Immunother. 2020 Oct 17;70(3):857–68. doi: 10.1007/s00262-020-02738-x (PMC7907015; doi:10.1007/s00262-020-02738-x)
Supplement: Supplementary file 1 — Supplementary material 1 (DOCX 21 kb) [file 262_2020_2738_MOESM1_ESM.docx]

**Supplementary material**

**PD-L1 IHC 22C3 assay**

Formalin Fixed Paraffin Embedded tissue (FFPE) sections were collected from eligible patients at baseline. Sections were stained with the primary antibody of anti–PD-L1 28-8 rabbit monoclonal antibody or anti–PD-L1 22C3 mouse monoclonal antibody, using Envision FLEX visualization system on Autostainer Link 48 (clone 22C3, Dako, Carpinteria, CA). The control cell line slide containing the PD-L1 positive & PD-L1 negative cell lines (provided in the Dako Kit) were stained with each batch of specimens, as described in the PD-L1 IHC 22C3 pharmDx Interpretation Manual (<https://www.agilent.com/cs/library/usermanuals/public/29158_pd-l1-ihc-22C3pharmdx-nsclc-interpretation-manual.pdf>).

**Tumor mutation burden analysis**

***Library construction and sequencing***

Tumor DNA was extracted from FFPE specimens with the black PREP FFPE DNA Kit (Analytik Jena AG, Jena, Germany) according to the manufacturer’s instructions. DNA concentration was measured using Qubit dsDNA HS Assay kit or Qubit dsDNA BR Assay kit (Life Technologies, California, USA) according to the manufacturer’s recommended protocol.

Tumor DNA and matched genomic DNA were sheared into 150-200-bp fragments by a Covaris M220 Focused-Ultrasonicator (Covaris, Massachusetts, USA). Fragmented DNA libraries were constructed with a KAPA HTP Library Preparation Kit (Illumina Platform) (KAPA Biosystems, Massachusetts, USA) according the manufacturer’s instruction. DNA libraries were captured with a designed gene panel, NimbleGen SeqCap EZ Library (Roche, Wisconsin, USA), that includes major tumor-related genes. The captured samples were then subjected to Illumina HiSeq X-Ten for paired-end sequencing.

***Identification of somatic mutations***

FFPE tumor and matched blood cell samples were submitted for next generation sequencing. We used VarScan2 with the following filters: (1) located in intergenic regions or intronic regions; (2) synonymous SNVs; (3) allele frequency ≥0.002 in the genome Aggregation Database (gnomAD) database; (4) allele frequency <0.05 in the tumor sample; (5) allele depth <5 and reads in each strand equals 0; and (6) the value of ljb2_pp2hdiv="B" and ljb2_pp2hvar="B"; (7) the value of snp138 and cosmic68 both not equal to “.”. In order to identify somatic SNV and indel mutations, the mutations in FFPE tumor samples were blanked by matched blood cell samples from patients respectively; if blood cell samples were not supplied, the mutations of allele frequency >0.4 in FFPE tumor samples were dropped.

***Calculation of tumor mutational burden***

For the determination of FFPE tumor samples’ tumor mutational burden (TMB) value, mutation sites are remained using following criterions with aboved snv results: (1) nonsynonymous SNVs; (2) allele frequency<0.002 in the Exome Aggregation Consortum (ExAC) database; (3) depth of allele >=100; Alterations likely or known to be bona fide oncogenic drivers were excluded. The depth of each allele in the region of panel were calculated with samtools and excluded the allele that depth lower than 100. TMB was measured in mutations per Mb.

**Quantitative T cell repertoire with sequencing**

***PBMC preparation and CD8^+^ T cells isolation from enrolled patients***

Samples of peripheral blood mononuclear cells (PBMC) were collected from 11 patients. PBMCs were immediately isolated from each patient using Ficoll-Hypaque 1077 (Sigma-Aldrich) gradient centrifugation. Total CD8^+^ T cells were enriched from PBMCs with the BD IMag™ Anti-Human CD8 Magnetic Particles kit (Catalog No. 557766).

***TCR β amplification and sequencing***

RNA extraction of total CD8^+^ T cells was performed using RNeasy Plus Mini Kit (Qiagen, Valencia, CA, USA) based on the manufacture’s instruction.

One common forward primer adaptor and one reverse primer corresponding to the constant (C) regions of each of the *TCR β* were designed to facilitate PCR amplification of cDNA sequences in a less biased manner. Samples were analyzed by high-throughput sequencing of TCR using the ImmuHub^®^ TCR profiling system at a deep level (ImmuQuad Biotech, Hangzhou China). Briefly, a 5’ RACE unbiased amplification protocol was used. Sequencing was performed on an Illumina HiSeq^®^ system with PE150 mode (Illumina). One common adaptor was added on the 5’ of cDNA during the first-strand cDNA synthesize and one reverse primer corresponding to the constant (C) regions of each of the *TCR β* were designed to facilitate PCR amplification of cDNA sequences in a less biased manner.

To estimate *TCR β* clonotype diversity, Shannon’s Index of Diversity (H’) was calculated according to the formula:

H’=-∑pi(ln pi)

Where n is the total number of *TCR β* clonotypes and pi is proportion of clonotype i in the *TCR β* repertoire.

The clonality index [1]specific for characterizing the situation of T cell clonal proliferation was calculated based on the following equation:

$$clonality=1-\frac{diversity}{\ln\left( productive uniques \right)}$$

Where productive uniques are the total number of clonotypes and diversity is calculated using the following formula:

$$Diversity=\sum\frac{pi(\ln pi)}{\ln S}$$

Where pi is the proportion of sequence i relative to the total sequences and S is total quantity of clonotypes.

***Statistics and graph***

Two tailed Student’s t-test was used for the comparison of Shannon’s diversity index, and clonality index between pre- and post-treatment groups, by GraphPad Prism version 6.0 (La Jolla, CA, USA). A *P*-value < 0.05 was considered statistically significant. The donut chart was drawn by R (Version 3.4.0).

**References**

1. Beausang JF, Wheeler AJ, Chan NH, Hanft VR, Dirbas FM, Jeffrey SS, Quake SR: **T cell receptor sequencing of early-stage breast cancer tumors identifies altered clonal structure of the T cell repertoire**. *Proceedings of the National Academy of Sciences of the United States of America* 2017, **114**(48):E10409-e10417.
